# Surgical Aspects of No-Touch Saphenous Vein Graft Harvesting in CABG: Clinical and Angiographic Follow-Up at 3 Months

**DOI:** 10.21470/1678-9741-2018-0352

**Published:** 2019

**Authors:** Ninos Samano, Bruno Botelho Pinheiro, Domingos Souza

**Affiliations:** 1 Department of Cardiothoracic and Vascular Surgery, Faculty of Medicine and Health, Örebro University, Örebro, Sweden.; 2 University Health Care Research Center, Faculty of Medicine and Health, Örebro University, Örebro, Sweden.; 3 Department Cardiovascular Surgery, Hospital do Coração Anis Rassi, Goiânia, GO, Brazil.

**Keywords:** Coronary Artery Bypass, Saphenous Vein, Vascular Patency, Mammary Arteries

## Abstract

With more than 800,000 coronary artery bypass grafting (CABG) operations annually worldwide and the saphenous vein being the most common conduit used, there is no question that improving saphenous vein graft patency is one of the most important tasks in CABG. This video describes the no-touch harvesting procedure of the saphenous vein on an 80-year old man with hypertension, hyperlipidemia and a previous myocardial infarction with percutaneous coronary intervention to the right coronary artery. He was complaining of exertional chest pain and was diagnosed with stable angina pectoris. The coronary angiography showed advanced three vessel disease with significant stenoses in the left anterior descending (LAD) artery, two marginal arteries (MAs) and the posterior descending artery (PDA), in addition to an occluded diagonal artery (DA). The patient received a triple sequential no-touch vein graft to the PDA and two MAs together with a double sequential no-touch vein graft to the DA and LAD. A vein graft was used to bypass the LAD due to the age of the patient and the low degree of stenosis in the LAD. The no-touch harvesting technique is described in detail in the film with complete narration. A follow-up of this patient was performed at three months both clinically and with a computed tomography angiography (CTA). No angina pectoris symptoms were reported by the patient and the wounds in the chest and lower limb were completely healed. The CTA showed patent no-touch saphenous vein grafts to all the distal anastomoses.

**Table:** 

Abbreviations, acronyms & symbols	
CABG	= Coronary artery bypass grafting
CTA	= Computed tomography angiography
DA	= Diagonal artery
LAD	= Left anterior descending
MAs	= Marginal arteries
PDA	= Posterior descending artery

## PATIENT CHARACTERISTICS

Eighty-year-old man with hypertension, hyperlipidemia and a previous myocardial infarction with percutaneous coronary intervention to the right coronary artery. The ejection fraction was 55% and he was complaining of exertional chest pain. The coronary angiography showed advanced three-vessel disease with significant stenoses in the left anterior descending (LAD) artery, two marginal arteries (MAs) and the posterior descending artery (PDA), in addition to an occluded diagonal artery (DA). The patient received a triple sequential no-touch vein graft to the PDA and two MAs together with a double sequential no-touch vein graft to the DA and LAD. A vein graft was used to bypass the LAD due to the age of the patient and the low degree (60-70%) of stenosis in the LAD.

## SURGICAL TECHNIQUE

The no-touch technique in harvesting the saphenous vein consists of several steps in order to get a good quality conduit as well as to reduce leg wound complications (Video 1). Previous studies have shown a superior patency for the no-touch vein grafts in both the short- and long-term^[1-4]^.

**Video 1:**
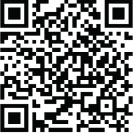
Surgical technique of no-touch saphenous vein graft harvesting in coronary artery bypass grafting.

### Vein Mapping

Preoperative vein mapping facilitates rapid and accurate location of the vein, thus reducing soft tissue injury and the creation of tissue flaps. It also helps to predetermine the best segment of the vein for grafting.

### Exposure

An incision is made through the skin and the saphenous vein is exposed by lifting the edges of the skin. This enables the identification of the correct dissection plane. The diathermy knife can consequently be used safely. The subdermal vessels are ligated to avoid overuse of diathermy.

### Marking of Pedicle

The pedicle is marked with a diathermy knife approximately 0.5 cm from the both sides of the vein. The large vasa vasorum and the small side branches are ligated proximally with clips. On the other hand, larger side branches should be ligated with a suture.

### Removal from Bed

**Fig. 1:**
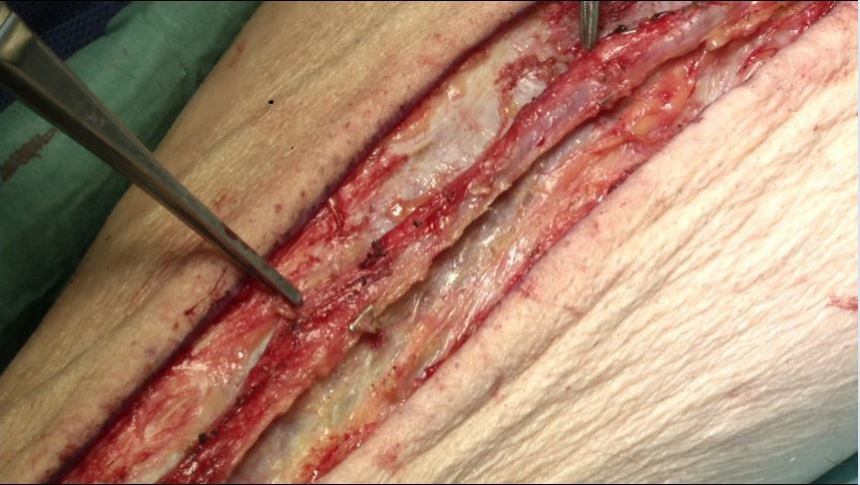
No-touch vein graft in situ.

The saphenous vein is removed from its bed using both scissors and a diathermy knife. The same dissection plane is maintained along the whole length of the graft (Figure 1). After removal, the vein is stored in heparinized blood.

### Closure of the Wound

The wound should be closed in two to three layers. The first layer is especially important to avoid the creation of a dead space where the fascia is usually included in the suture. The second layer is to bring the edges together without tension in the skin. The third layer is with a continuous intracutaneous suture. We recommend performing two separate incisions excluding the knee area as this is more convenient for the patient.

### Checking for Leakage

After starting cardiopulmonary bypass and before cross clamping the aorta, the vein graft is connected to the arterial line and any unligated side branches can be identified. The end of the graft is then prepared for the distal anastomoses. This can be achieved in off-pump surgery by performing the proximal anastomsis first. Only one side of the vein is dissected. This allows a simple grasping of the pedicle without direct manipulation of the vein.

### Distal Anastomosis

The perivascular tissue is used to grasp the vein, giving a good exposure during the suturing. After each complete distal anastomosis, the vein is briefly connected to the arterial line to check for leakage. Finally, the pedicle is fixed to the epicardium at the level of the anastomosis. The no-touch vein simplifies the use of sequential grafts as kinking is not an issue.

### Central Anastomosis

After release of the cross-clamp, the grafts are now connected to the arterial line to allow early myocardial perfusion. This also helps to determine graft length, recheck for bleeding and to maintain relaxation of the vein. The central anastomoses are performed in a classic fashion. A final inspection of the grafts before weaning off cardiopulmonary bypass is also recommended.

### Flow Measurement

Flow measurement with a transonic coronary flow probe is always performed after weaning off cardiopulmonary bypass.

**Table:** 

Authors' roles & responsibilities	
NS	Substantial contributions to the conception or design of the work; or the acquisition; drafting the work or revising it critically for important intellectual content; final approval of the version to be published
BBP	Substantial contributions to the conception or design of the work; or the acquisition; drafting the work or revising it critically for important intellectual content; final approval of the version to be published
DS	Substantial contributions to the conception or design of the work; or the acquisition; drafting the work or revising it critically for important intellectual content; final approval of the version to be published
